# Effects of a natural polyphenol on nicotine -induced pancreatic cancer cell proliferation

**DOI:** 10.1186/1617-9625-12-S1-A7

**Published:** 2014-06-06

**Authors:** Parimal Chowdhury, John JayRoe

**Affiliations:** 1 Department of Cellular Physiology and Molecular Biophysics, University of Arkansas for Medical Sciences, Little Rock, Arkansas 72205, USA

## Background

Resveratrol (3, 5, 4’-trihydroxy-transstilbene), a phytoalexin derived from the skin of grapes and other fruits is perhaps cancer chemo-preventive. It is known to have potent anti-inflammatory and anti-oxidant effects and inhibit platelet aggregation and the growth of a variety of cancer cells. In vitro and in vivo studies have confirmed that resveratrol can modulate multiple pathways involved in cell growth, apoptosis, and inflammation. Its anti-carcinogenic effects appear to be closely associated with its antioxidant activity, and thus the use of resveratrol in human cancer chemoprevention, provide a rationale in support of its use. However it is not known, whether or not it provides the inhibitory effects on proliferative pancreatic cancer cells induced by nicotine.

## Materials and methods

In this study we have examined the effects of resveratrol on nicotine induced pancreatic cancer cell proliferation. Two different cultured pancreatic cancer cell lines were used in this study. Cell proliferation was examined in a dose and time dependent manner by MTT assay and its mechanism was further examined through mitogen activated protein kinase signal transduction pathways (MAPK) employing ERK 1 and ERK 2 antibodies. The data with respect to its inhibitory effects obtained with Western Blot and immunohistochemical co-localization methods were analyzed and compared between these two cell lines. The dose of resveratrol selected for the study was determined from an earlier study.

## Results

The results show that both of the pancreatic cancer cell lines are susceptible to inhibitory effects of this compound. However their relative effects differed. The proliferative effects of these cultured cells in response to nicotine appeared to have been mediated by oxidant activates and these effects were reversed by this dose of resveratrol.

## Conclusions

We conclude from our study that the anti-carcinogenic effects of resveratrol are closely associated with its antioxidant activity in both human and rat pancreatic tumor cells. Further studies will be conducted to determine the chemo preventive role of this compound in transgenic pancreatic cancer model.

